# Large-scale genomic analyses reveal alterations and mechanisms underlying clonal evolution and immune evasion in esophageal cancer

**DOI:** 10.1038/s41467-023-36557-2

**Published:** 2023-02-17

**Authors:** De-Chen Lin

**Affiliations:** 1 Center for Craniofacial Molecular Biology, Herman Ostrow School of Dentistry, University of Southern California, Los Angeles, CA USA; 2 Norris Comprehensive Cancer Center, University of Southern California, Los Angeles, CA USA

**Keywords:** Oesophageal cancer, Cancer genomics, Tumour heterogeneity, Tumour immunology, Cancer microenvironment

## Abstract

Esophageal cancers feature distinct manifestations between and within patients which complicate precision diagnosis, prognosis, and patient care. New genomic and epigenomic research uncovers novel mechanisms underlying both inter- and intra-tumoral heterogeneity in esophageal cancer, with significant biological and translational implications.

Esophageal squamous cell carcinoma (ESCC) is a common and lethal malignancy worldwide. A number of tumor sequencing studies have hitherto established the genomic landscape of ESCC; however, these efforts have led to neither genome-guided targeted therapies nor molecular-based diagnostic or prognostic biomarkers. Instead, genomic analyses have underscored highly heterogeneous genomes of ESCC, manifesting in both inter- and intra-tumoral forms^1^. Inter- and intra-tumoral heterogeneity are both recognized to strongly affect cancer diagnosis, prognosis and clinical management, thereby contributing to treatment failure and poor clinical outcome.

The ESCC genome exhibits high inter-tumoral heterogeneity and yet moderate-to-low mutational burden, posing a key challenge for comprehensively identifying genomic drivers and actionable mutations. In fact, based on the background mutational rate of ESCC, saturation analyses estimated that identification of driver mutations in 2–3% of the patients requires sequencing of 1500-2000 tumor genomes^2^. The high inter-tumoral heterogeneity and long-tailed mutation list also suggest that most of genomic drivers and druggable mutations are present only in a small fraction of ESCC tumors, an obvious obstacle for implementing the panel-sequencing approach for patient stratification and selection. Intra-tumoral heterogeneity fuels cancer evolution and diversification, causing immune evasion, drug resistance and disease recurrence. Moreover, intra-tumoral heterogeneity often leads to sampling bias, hindering precision diagnosis and personalized medicine.

Clinically, ESCC tumors are often resistant to cytotoxic chemoradiotherapy; ESCC patients suffer from dismal outcomes, having a 5-year survival rate lower than 20%^3^. Undoubtedly, an urgent need exists to characterize further ESCC genomes for the development of personalized treatment and oncological biomarkers. This requires not only sequencing a large number of patients to overcome its strong inter-tumoral heterogeneity, but also characterizing in detail the clonal makeup and evolution to decipher ESCC intra-tumoral heterogeneity. New research^4,5^ published recently in *Nature Communications* have specifically taken on these two challenges, by performing genomic and epigenomic analyses with significant breadth and depth, and shed novel insights into ESCC tumor heterogeneity with translational implications.

To address the inter-tumoral heterogeneity, Li et al.^5^ curated and integrated ESCC sequencing data from 1930 patients across 33 studies, representing one of the largest compendia of paired tumor-normal sequences. To ensure data quality and minimize batch effects, the authors paid extra attention and made efforts during quality control, data processing, homogenization and verification. The large number of sequencing data revealed several new significantly mutated genes in ESCC, including *PPFIA2, TGFBR2, ZBBX, ATP13A5* and *IRF2BPL*. In addition, novel recurrent frameshift deletions in *TGFBR2* and *IRF2BPL* were identified, indicative of their tumor suppressing functions. The collection of clinicopathological and epidemiological information of most sequenced patients, coupled with high statistic power, enabled robust clinical associations. For example, *NOTCH1* mutations were prominently associated with the increased diagnostic age of ESCC patients, consistent with previous findings of *NOTCH1* genomic alterations in normal esophageal tissues of elderly individuals^6^. Both log rank test and multivariable adjusted Cox analysis revealed gene mutations associated with inferior prognosis in both early- and late-stage patients. On the other hand, certain gene mutations were associated with stage-specific survival. For example, *NFE2L2* mutations were specifically correlated with poor survival of late-stage patients. This association is likely explained by prior findings that *NFE2L2* mutations conferred chemotherapy and radiation resistance in ESCC cells^7^.

Nevertheless, most of the mutations with prognostic values occurred in less than 5% ESCC patients, limiting their potential clinical application as biomarkers individually. The authors thus built a statistical model to establish a gene-panel-based mutational score for prognosis prediction. Upon balancing the positive rate and the complexity of the model, an eight-gene mutational score was established, defined as the sum of nonsynonymous mutations *in NFE2L2, CSMD1, CREBBP, KALRN, PRUNE2, NRXN1, AKAP9* and *FREM2*. In early-stage patients, 1-2 and >2 positive mutations were respectively associated with 1.78 and 2.26 of hazard ratio (HR), indicating substantial predictive power. A similar result was found in late-stage patients. The prognostic property was validated in multiple independent datasets upon stage adjusted HR in Cox regression, confirming the mutational score as a robust prognostic model in ESCC. Further facilitating its potential clinical use, the eight-gene panel was able to cover a considerable proportion of ESCC patients, with 29.1% early- and 27.8% late-stage patients having at least one mutation. This detection rate substantially overcomes the challenge of inter-tumoral heterogeneity of ESCC patients.

In the other study^4^, Cui and colleagues performed one of the largest analyses of intra-tumoral heterogeneity of ESCC, characterizing 186 primary samples from 36 patients. Multi-omic sequencing, including whole exome sequencing (WES), reduced representation bisulfite sequencing (RRBS) and RNA-seq were performed on the majority of samples. This comprehensive strategy enables in-depth analyses of genomic, epigenomic and transcriptomic intra-tumoral heterogeneity and the interplay between these mechanisms during tumor evolution, addressing several key questions.

Perhaps one of the most intuitive questions is what are the molecular mechanisms driving intra-tumoral heterogeneity at different molecular levels. The study provided compelling evidence showing that chromosomal instability is the most significant force promoting both genomic and epigenomic intra-tumoral heterogeneity. Indeed, genome doubling was associated with subclonal copy-number alterations. Epigenomically, somatic copy number altered regions showed a higher degree of epigenetic entropy and DNA methylation variability. At the transcriptomic level, genes with subclonal copy number gains had higher mRNA expression and the reverse was also true. Additionally, variable promoter methylation levels also contribute to the intra-tumoral expression heterogeneity.

It is unclear if genomic and epigenomic changes converge or diverge during tumor evolution and why. Different patterns have been reported regarding the phylogeny relationship between somatic mutations and DNA methylation in different cancer types. For example, in an earlier study of ESCC, the phyloepigenetic tree of DNA methylation changes followed exactly the tree of passenger mutations in the genome^8^. This is what one would expect from those loci where DNA methylation reflects a passive mitotic clock. However, the evolution trajectories of somatic mutations and DNA methylation were largely discordant in liver cancer^9^. This would be expected for methylation loci that are associated with active gene regulatory elements rather than a passive mitotic clock. Here^4^, both concordant and discordant patterns of phylogenetic trees were also noted in this large collection of intra-tumoral sequencing data. Clarifying these disparities thus warrants future investigation of the associations between mitotic turnover vs. gene regulation.

Another important question with translational significance is how the cross-talk between tumor cells and immune microenvironment influences cancer evolution. Relatedly, it is unclear what are the mechanisms promoting clonal and subclonal immune evasion. The authors^4^ first deconvoluted RNA-seq data, revealing diverse levels of immune infiltrates across different spatial regions within the same tumor, which was further validated by orthogonal multiplex immunostaining. The intra-tumoral variability of somatic mutations was associated with immune infiltration diversity, echoing a previous finding of a strong correlation between the repertoire of T cell receptors (a measurement of T cell clonality) and genomic intra-tumoral heterogeneity in ESCC^10^. Neoantigens were then predicted bioinformatically, and it was noted that intra-tumoral heterogeneity of neoantigens was stronger in tumors with more diverse immune infiltration, indicating the specific selective pressure on neoantigens by tumor-infiltrating lymphocytes. In probing the mechanisms underlying immune escape, the authors identified that out of 36 patients, nine harbored clonal loss of heterozygosity (LOH) of HLA and two with subclonal LOH, suggesting that LOH of HLA is an early and prevalent genetic mechanism behind immune evasion. In addition to copy number changes, mRNA expression of HLA was also interrogated. Importantly, intra-tumoral variability of infiltrating CD8^+^ T cells and NK cells were significantly correlated with the intra-tumoral expression heterogeneity of HLA-A, HLA-B, and B2M, suggesting that the intra-tumoral expression heterogeneity of HLA genes is driven by and adaptive to diverse immune cell attacks.

In summary, by performing large-scale genomic investigation, combined with in-depth computational and statistical analyses, these new studies^4,5^ have uncovered novel mutational features exhibiting robust associations with key clinical parameters, revealed molecular mechanisms driving clonal diversification, and characterized the interplay between immune selection pressure and tumor evolution. Moving forward, new single-cell genomic and epigenomic technologies are revolutionizing the research on tumor heterogeneity, and recent single-cell RNA-seq studies^11–13^ have begun to highlight the extensive diversity of gene expression programs among ESCC cancer cells. For example, prominent inter- and intra-tumoral expression heterogeneity at both individual gene level and the pathway level were conspicuous in these single-cell datasets^11–13^. Moreover, certain gene expression programs from subsets of tumor cells were associated with the variability of immune infiltration, indicating the cross-talk between diverse clusters of malignant cells and immune populations^13^. The future development of methodologies to profile somatic mutations, chromatin accessibility and DNA methylation from the same cells will provide an exceptional opportunity to investigate the relationship between phylogenetic and phyloepigenetic architectures during cancer evolution. Moreover, the status quo of tumor heterogeneity research remains static and descriptive, with a clear gap between tumor sequencing and functional annotation of cancer genome and epigenome. One kind of models that would allow for such functional interrogation in a versatile and robust manner are patient-derived tumor organoid cultures. In fact, organoid modeling has been successfully applied to investigate both inter-^14^ and intra-tumor^15^ heterogeneity in several cancer types. The exciting new technologies, combined with advanced theoretical understanding of tumor heterogeneity, will significantly accelerate the development of precision diagnosis, prognosis, and personalized care of ESCC patients.

## Reporting summary

Further information on research design is available in the Nature Portfolio Reporting Summary linked to this article.

## Supplementary information

Reporting Summary
